# Supplementary material for: Regulation of acetyl-CoA biosynthesis via an intertwined acetyl-CoA synthetase/acetyltransferase complex
Source: Nat Commun. 2025 Mar 15;16:2557. doi: 10.1038/s41467-025-57842-2 (PMC11910552; doi:10.1038/s41467-025-57842-2)
Supplement: Supplementary file 1 — Supplementary Information [file 41467_2025_57842_MOESM1_ESM.pdf]

Supplementary information for:

Regulation of acetyl-CoA biosynthesis via an intertwined acetyl-CoA synthetase/acetyltransferase complex.

Liujuan Zheng[1,2,5,6], Yifei Du[3,5], Wieland Steinchen[2], Mathias Girbig[1], Frank Abendroth[2], Ekaterina Jalomo-Khayrova[2], Patricia Bedrunka[2], Isabelle Bekeredjian-Ding[4], Christopher-Nils Mais[2], Georg Hochberg[1], Johannes Freitag[2], Gert Bange[1,2,6]

[1]Max-Planck Institute for Terrestrial Microbiology, Karl-von-Frisch Strasse 14, 35043 Marburg, Germany

[2]University of Marburg, Center for Synthetic Microbiology (SYNMIKRO) & Department of Chemistry and Biology, Karl-von-Frisch Strasse 14, 35043 Marburg, Germany

[3]MRC Laboratory of Molecular Biology, Cambridge CB2 0QH, UK.

[4]University of Marburg, Faculty of Medicine, Karl-von-Frisch Strasse 14, 35043 Marburg, Germany

[5]These authors contribute equally to this work.

[6]Corresponding author: Liujuan.Zheng@mpi-marburg.mpg.de, gert.bange@synmikro.uni-marburg.de

The file contains:

Supplementary Fig. 1-16

Supplementary Tables 1-4

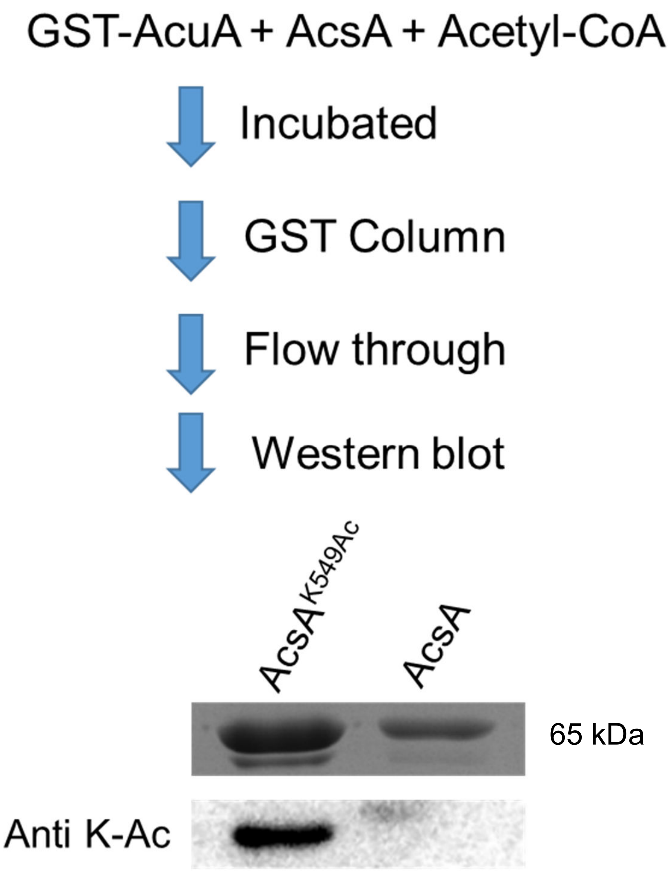

Supplementary Fig. 1. Western blot for the released AcsA from the AcsA-AcuA complex in the presence of Ac-CoA. To prepare acetylated AcsA (AcsA_K549Ac), 10 mg of AcsA, 10 mg of GST-AcuA, and 2 mg of Ac-CoA were incubated in 10 ml SEC buffer at 37°C for 60 minutes. The mixture was then diluted to 40 ml with SEC buffer and passed through a 5 ml GST-column to remove GST-AcuA. The flow-through containing AcsA_K549Ac was collected and concentrated to 6.5 mg/ml for Western blot assays. For the Western blot, 8 µL of 0.5 mg/ml AcsA_K549Ac and AcsA samples were subjected to SDS-PAGE and transferred to a PVDF membrane (7 min, 1.3 A, 25 V). The membrane was blocked with 10% NFDM in 1x TBST for 1 hour at RT. It was then incubated overnight at 4°C with anti-acetyl-lysine antibodies (1:1500 in TBST/5% (w/v) NFDM), washed three times with TBST, and incubated for 1 hour at 4°C with anti-rabbit IgG-alkaline phosphatase antibodies (1:1500 in TBST/0.5% (w/v) NFDM). Signals were detected using the ECL prime system and documented with a Fusion-SL chemiluminescent imager (Peqlab). The experiment was repeated three times with similar results. Source data are provided as a Source Data file.

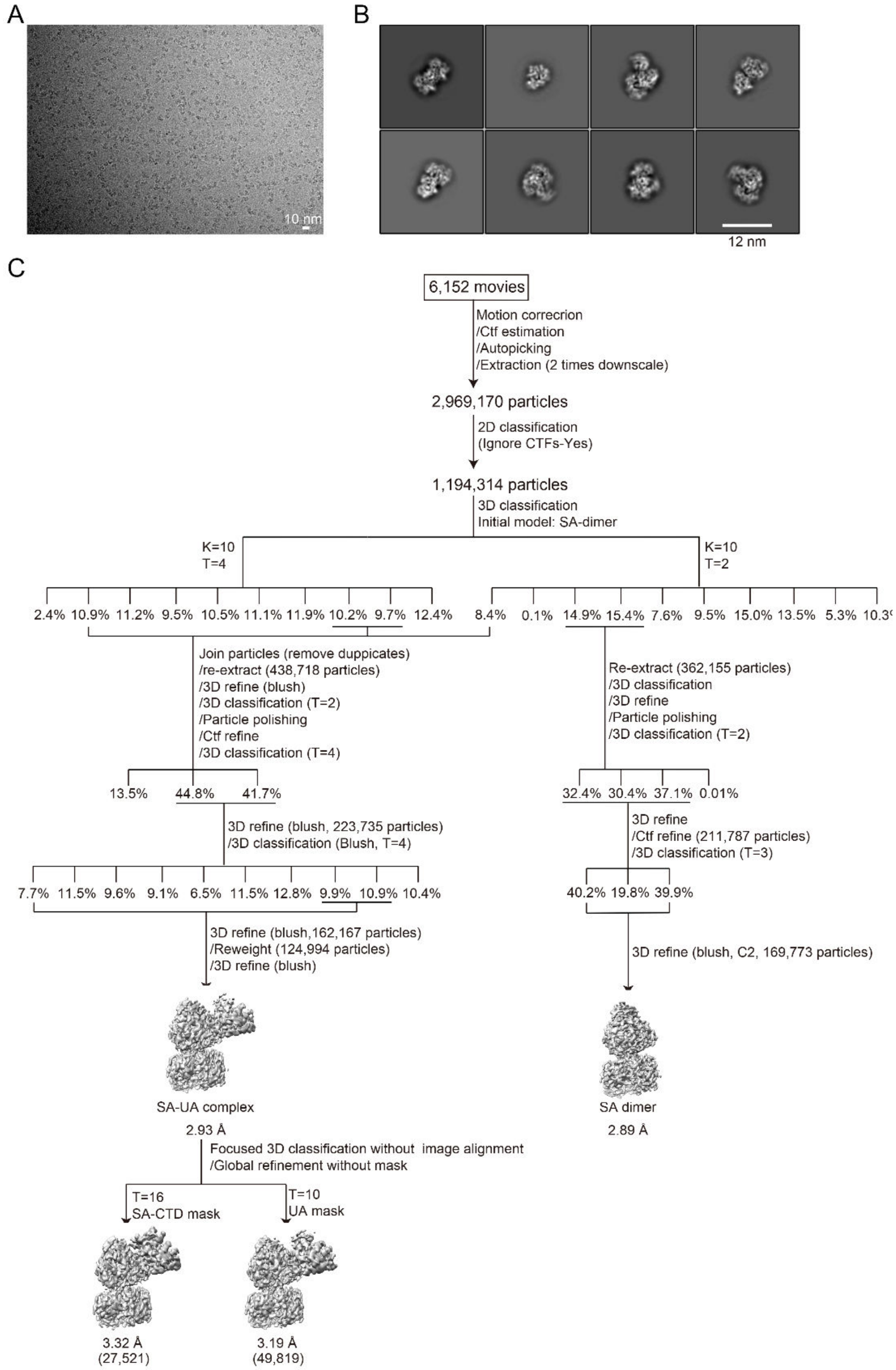

Supplementary Fig. 2. Data processing for AcsA/AcuA complex. **A** Representative cryo-EM micrograph. Scale bar = 10 nm. **B** Selected 2D class averages. Scale bar = 12 nm. **C** Flowchart of 3D classification and reconstruction.

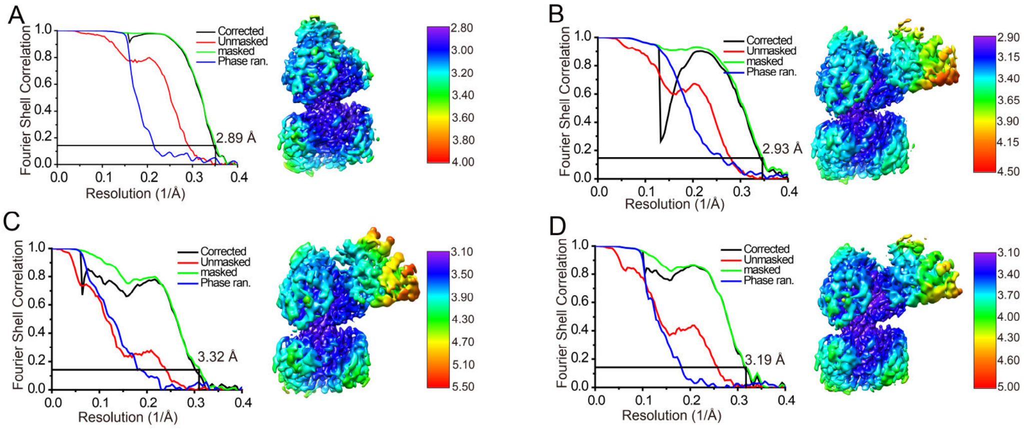

Supplementary Fig. 3. Resolution estimation of AcsA dimer and AcsA2-AcuA1 complex.

A-D. Fourier Shell Correlation curves (left panels) and cryo-EM maps coloured by local resolution values (right panels). **A.** AcsA dimer. **B.** AcsA2-AcuA1 complex. **C.** AcsA-CTD refined map. **D.** AcuA refined map. Overall resolution is estimated by FSC=0.143 criterion.

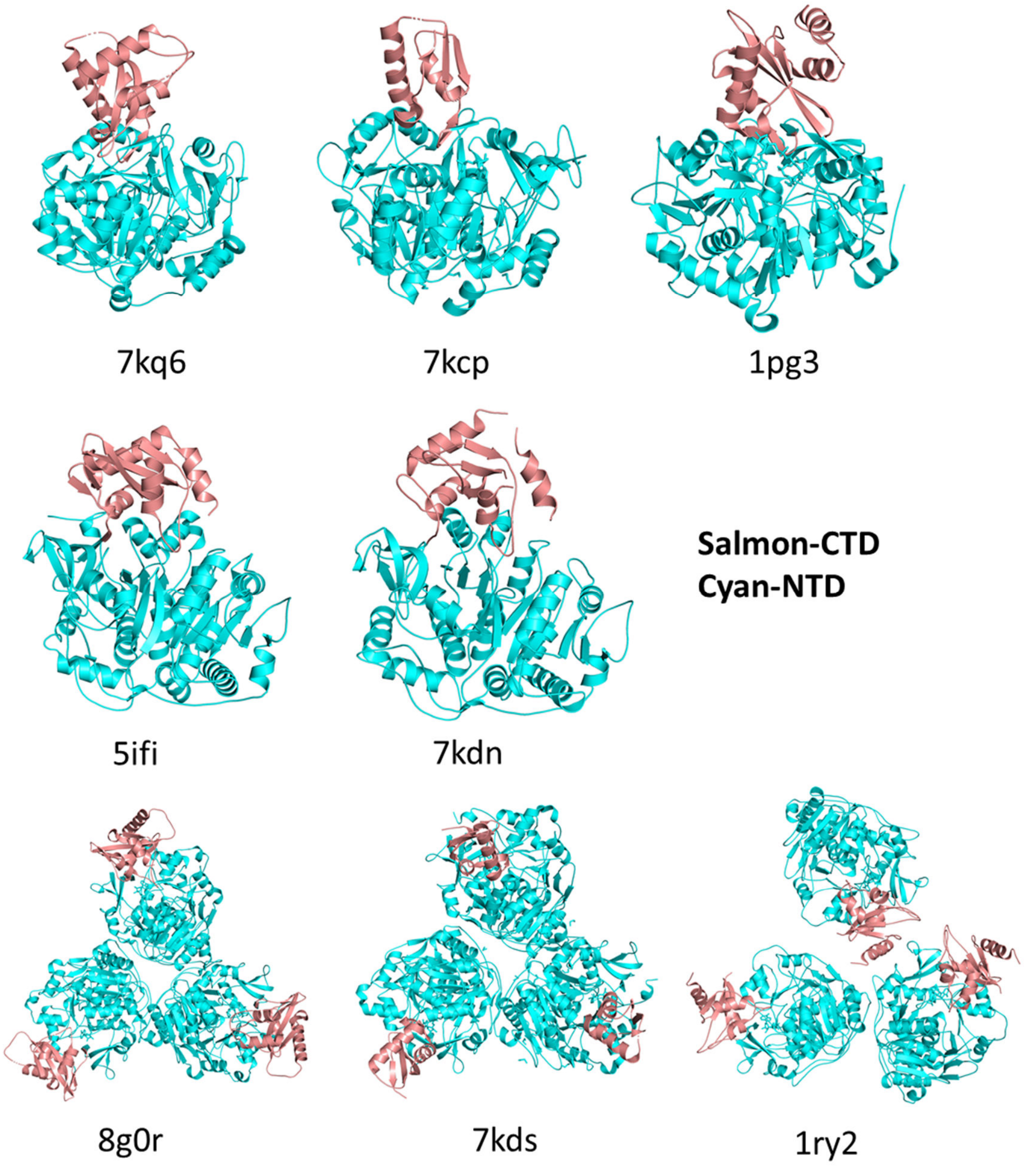

Supplementary Fig. 4. Some reported structures of Acs with different oligomerization status. Monomer Acs structures have been characterized from several organisms, including *Coccidioides immitis* (PDB: 7kq6), *Coccidioides posadasii C735* (PDB: 7kcp), *Salmonella enterica* (PDB: 1pg3)[1], *Cryptococcus neoformans var. grubii H99* (PDB: 5ifi), and *Aspergillus fumigatus* (PDB: 7kdn). In addition, trimer Acs structures have been identified from *Cryptococcus neoformans H99* (PDB: 8g0r), *Candida albicans* (PDB: 7kds), and *Saccharomyces cerevisiae* (PDB: 1ry2)[2], The C-terminal domain was colored salmon, while the N-terminal domain was colored cyan.

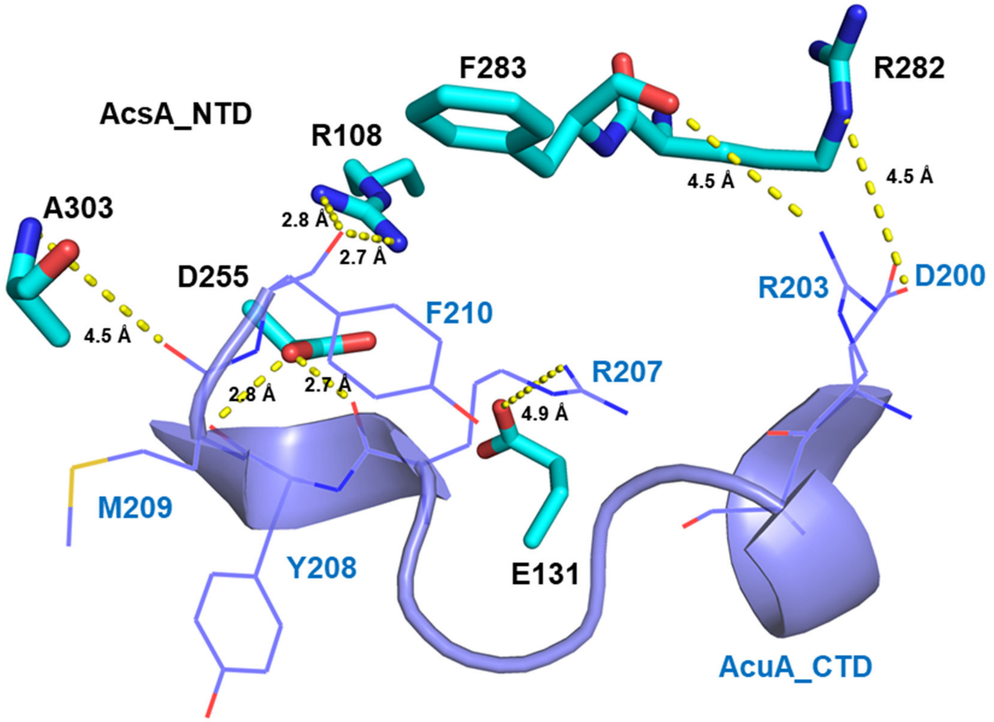

Supplementary Fig. 5. **C-terminus of AcuA bind with NTD of AcsA**. Detailed binding analysis of AcuA_CTD (depicted in blue cartoon and lines) to AcsA_NTD (represented by cyan sticks).

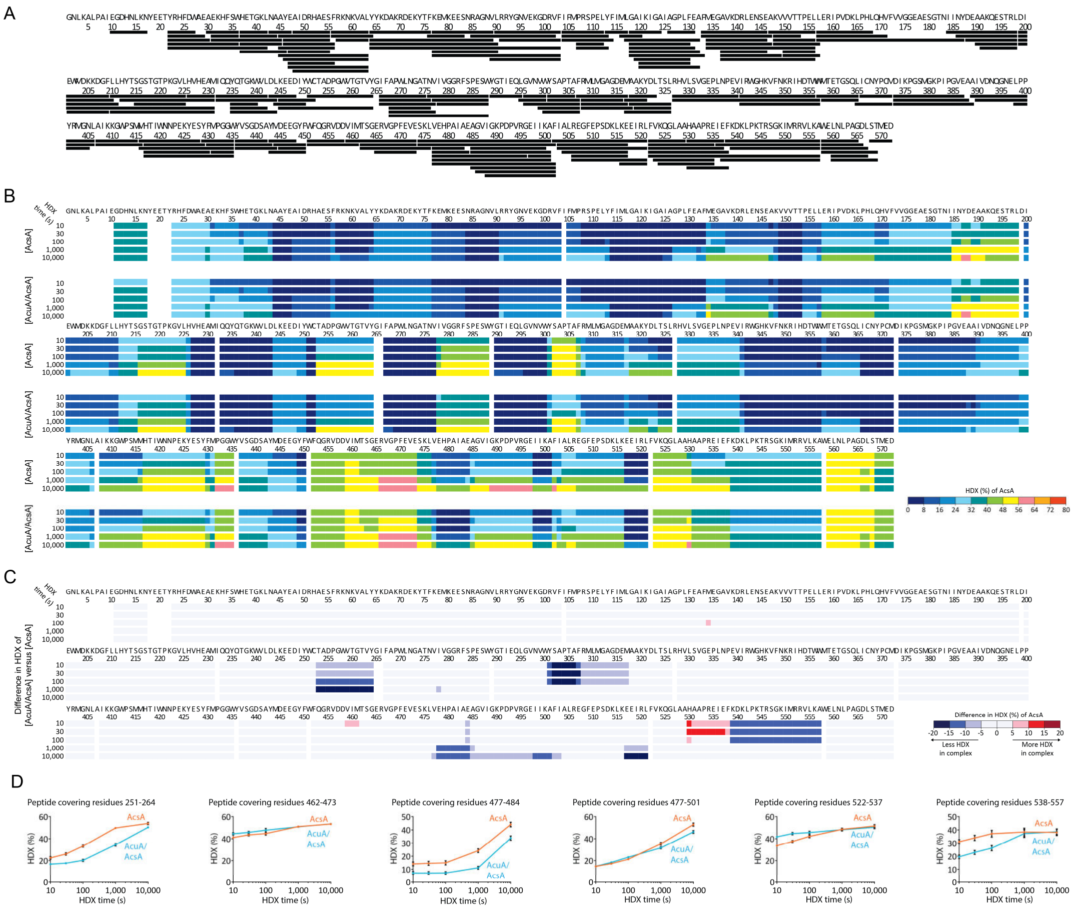

Supplementary Fig. 6. Interface of AcsA in the AcuA/AcsA complex by HDX-MS. **A**. Each black bar denotes an AcsA peptide identified by HDX-MS. **B**. Residue-specific HDX of AcsA, either in isolation or in context of the AcuA/AcsA complex. **C**. The difference in residue-specific HDX of AcsA when bound in the AcuA/AcsA complex versus AcsA alone. **D**. HDX of selected representative AcsA peptides. Data represent the mean ± s.d. of n = 9 replicates (three protein preparations measured in technical triplicates) are displayed.

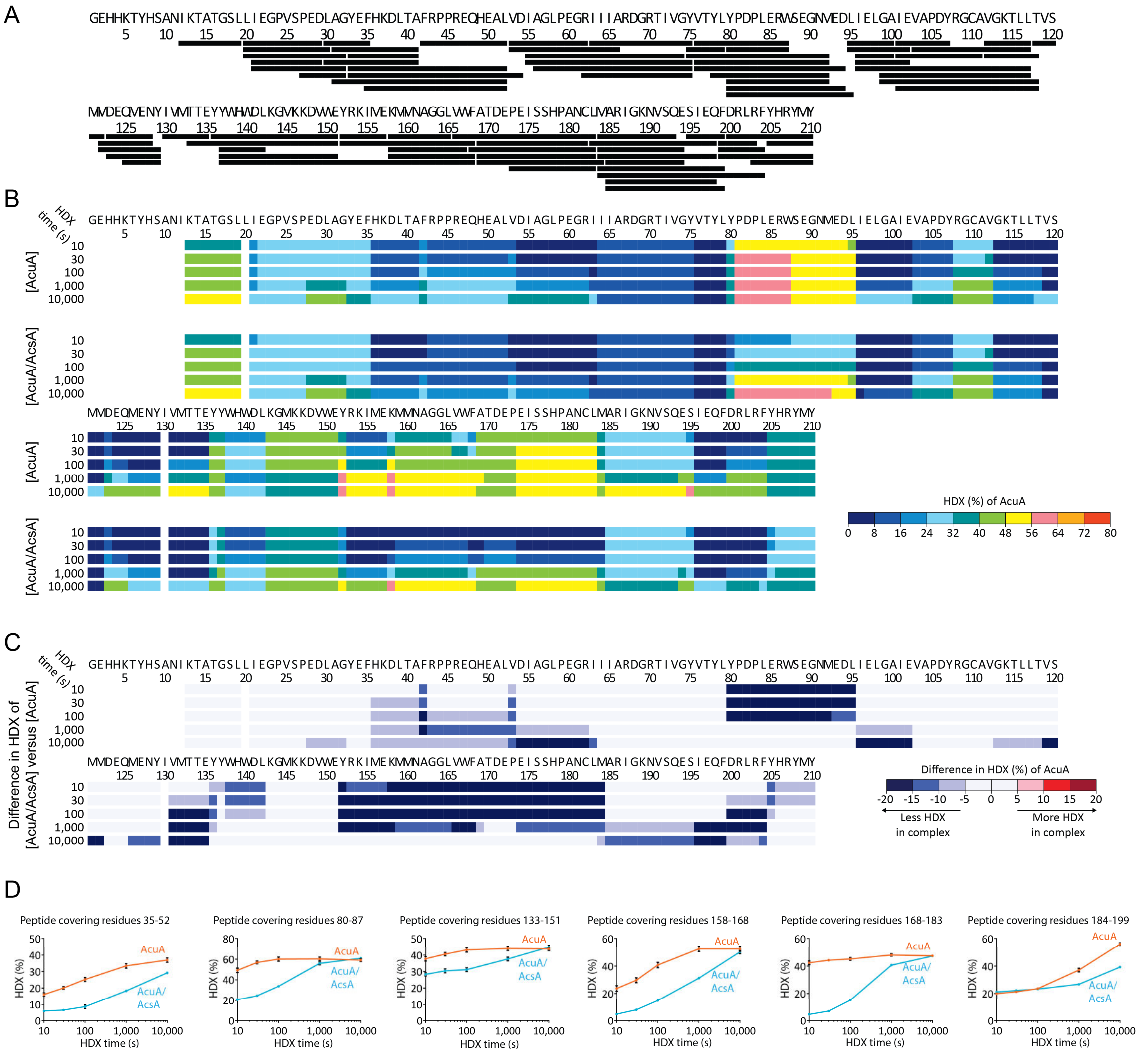

Supplementary Fig. 7. Interface of AcuA in the AcuA/AcsA complex by HDX-MS. **A**. Each black bar denotes an AcuA peptide identified by HDX-MS. **B**. Residue-specific HDX of AcuA, either in isolation or in context of the AcuA/AcsA complex. **C**. The difference in residue-specific HDX of AcuA when bound in the AcuA/AcsA complex versus AcuA alone. **D**. HDX of selected representative AcuA peptides. Data represent the mean ± s.d. of n = 9 replicates (three protein preparations measured in technical triplicates) are displayed.

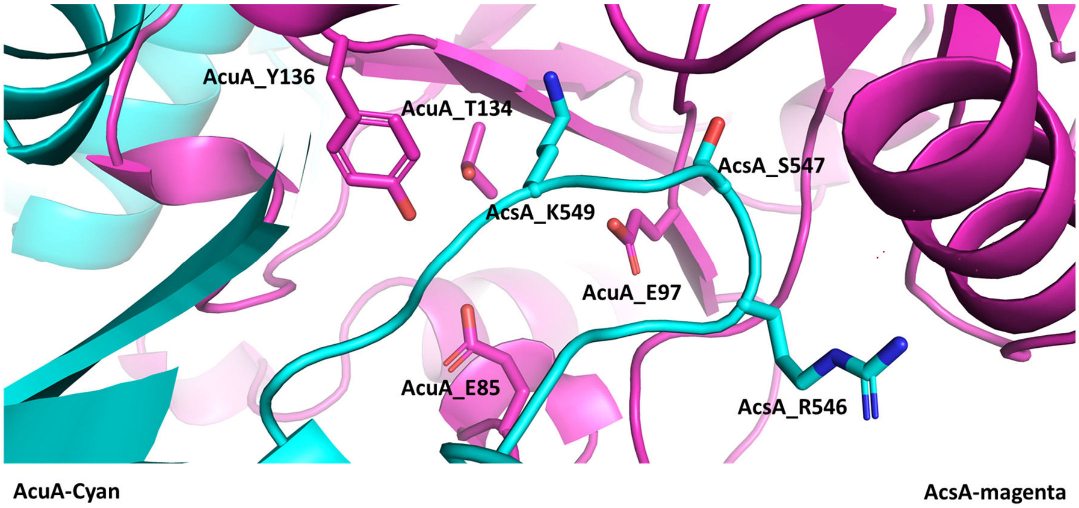

Supplementary Fig. 8. The interaction interface between the AcsA_K549 and the AcuA active site. Key residues at the interface are shown as stick representations: E85, E97, T134, and Y136 in AcuA (magenta) and R546, S547, and K549 in AcsA (cyan).

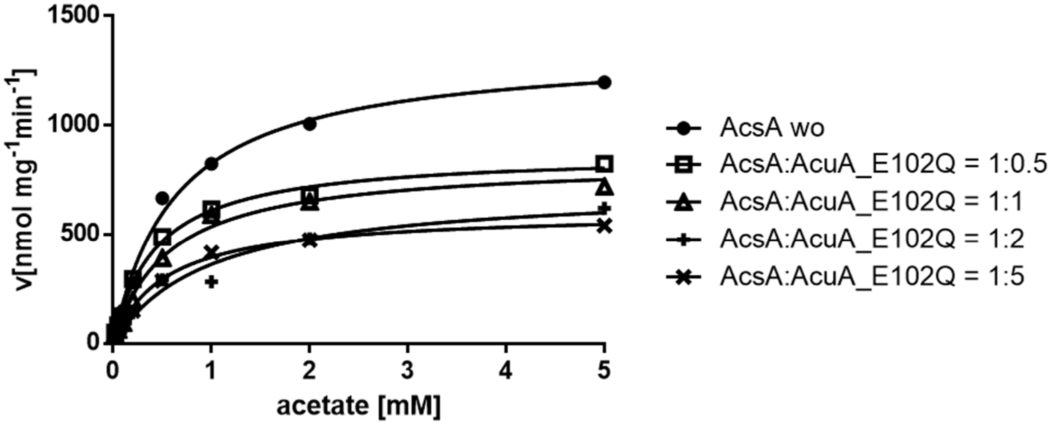

AcsA : AcuA_E102Q	V_{max} (nmol mg^{-1}min^{-1})	K_M (mM)
wo AcuA	1347	0.63
1:0.5	874	0.43
1:1	835	0.56
1:2	717	0.98
1:5	604	0.53

Supplementary Fig. 9. **Kinetic analysis of AcsA activity at varying AcuA_E102Q concentrations.** The data was fitted to a Michaelis–Menten curve using GraphPad software to determine the *Vmax* and *Km* values at different AcuA ratios. The experiment shows the mean of three technical replicates. Source data are provided as a Source Data file.

	Sequence		
12	H-RLRFYHRYMY-NH_2	Chemical Formula: $C_{71}H_{102}N_{22}O_{13}S$<br>Exact Mass: 1502.77<br>Molecular Weight: 1503.80	**m/z** **charge** **MW [Da]** **Error [Da]**<br>502.1 3+ 1503.2778257397 -0.053695710050079<br>752.7 2+ 1503.3852171598 0.053695710049851<br>**Deconvoluted MW [Da]: 1503.3315214498**<br>**Standard deviation [Da]: 0.075937201393914**

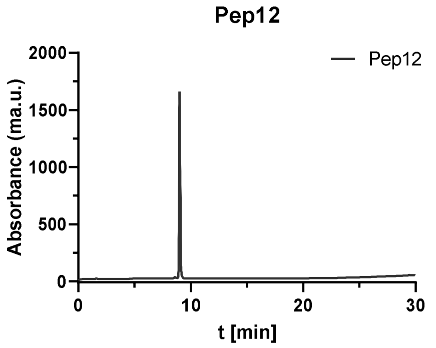

Supplementary Fig. 10. Chemical data of synthesized AcuA_CTD peptide. Sequence of peptide: RLRFYHRYMY. The chemical formula of the petide is: $C_{71}H_{102}N_{22}O_{13}S$, with a molecular calculated weight of 1503.80 g/mol. The synthesized peptide showed a molecular weight of 1503.33 g/mol.

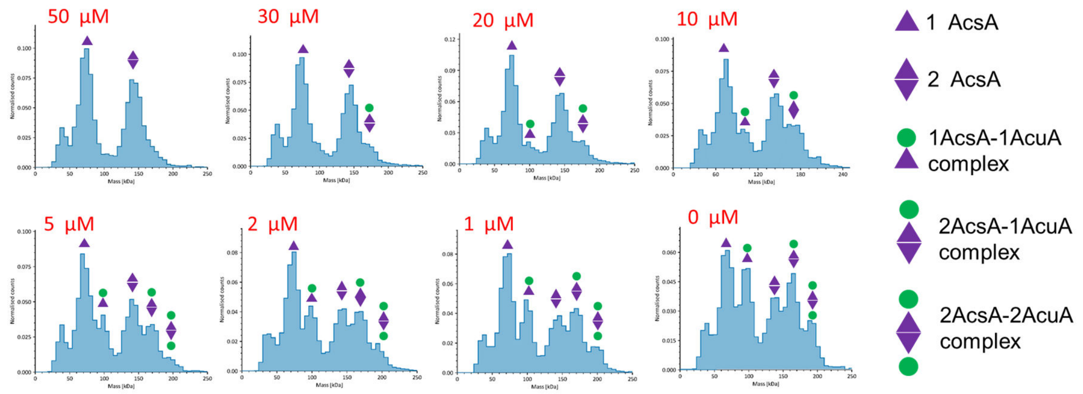

Supplementary Fig. 11. Influence of acetyl-CoA concentration to the AcsA-AcuA complex 37.5 nM AcuA and 37.5 nM AcsA were premixed, followed by the addition of Acetyl CoA. The mixture was then incubated for 1 minute before MP analysis.

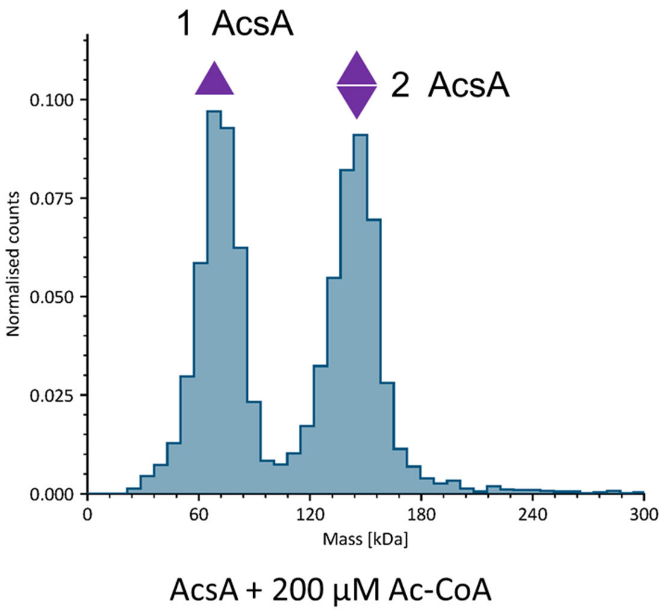

Supplementary Fig. 12. Influence of acetyl-CoA to the AcsA oligomerization status. 37.5 nM AcsA were premixed with of 200 µM acetyl-CoA. The mixture was then incubated for 1 minute before MP analysis.

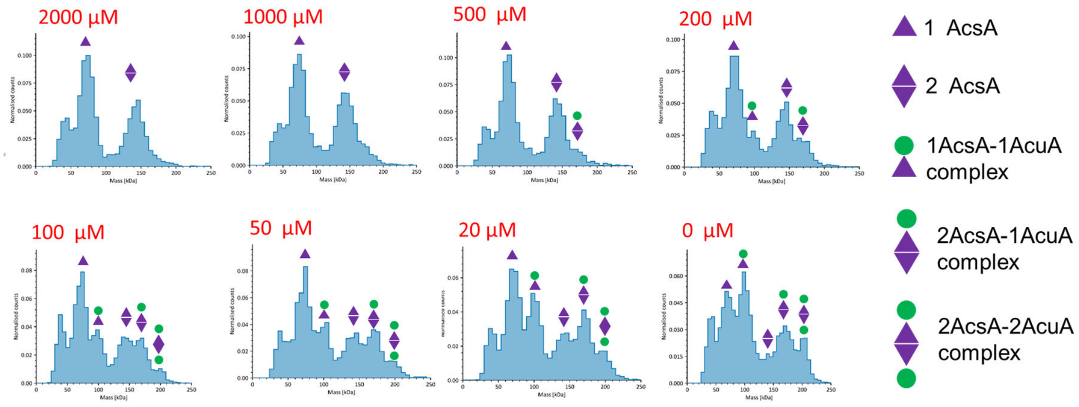

Supplementary Fig. 13. Influence of acetate concentration to the AcsA-AcuA complex. 37.5 nM AcuA and 37.5 nM AcsA, along with 500 µM ATP and 500 µM CoA, were premixed. Acetate was then added, and the mixture was incubated for 60 minutes before analysis.

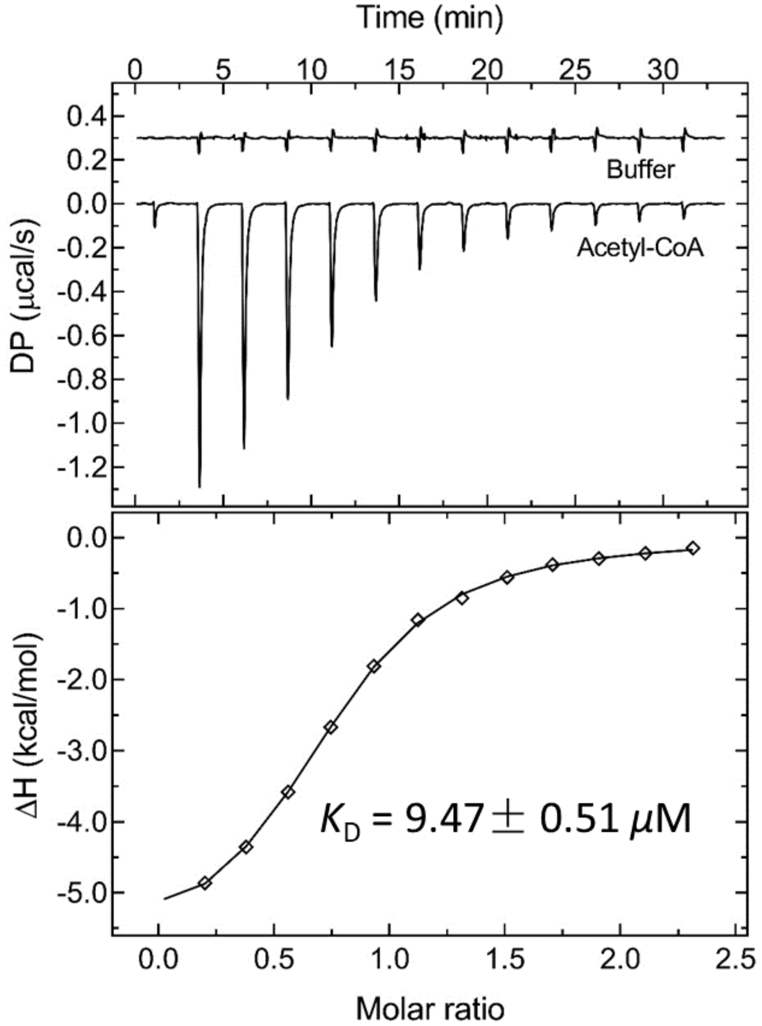

Supplementary Fig. 14. Binding affinity of acetyl-CoA to AcuA. Ligands and proteins were prepared in buffer (20 mM HEPES, 20 mM $MgCl_2$, 20 mM KCl, 200 mM NaCl, pH 7.5). BsAcua (81–83 µM) and Acetyl-CoA (1 mM) were analyzed using MicroCal PEAQ-ITC at 25 °C with 13 injections and 150 s spacing. The graph shows a representative experiment and the deviation is a result of the fitting. Source data are provided as Source data file. The experiment has been repeated twice with similar results.

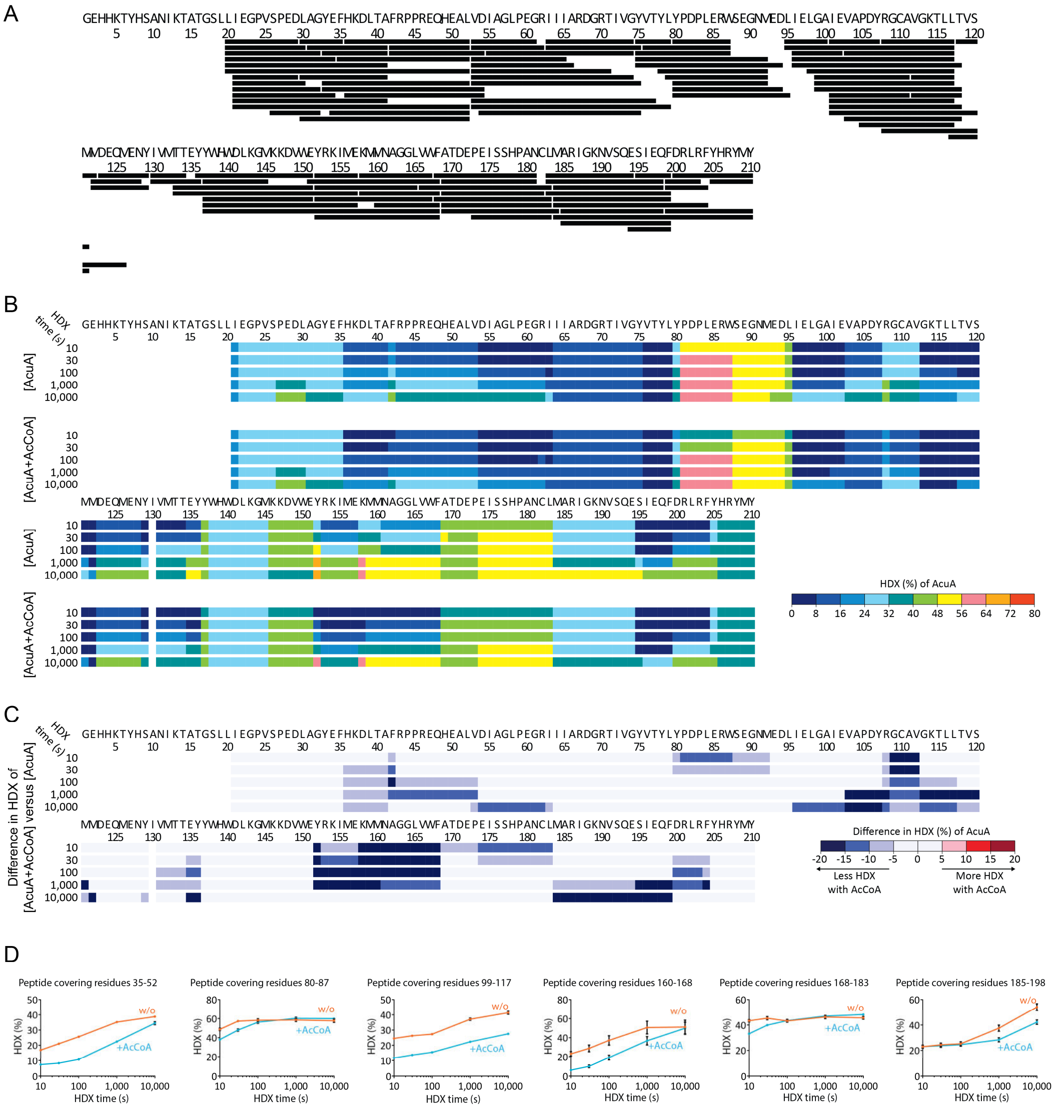

Supplementary Fig. 15. Acetyl-CoA binding to AcuA by HDX-MS. **A**. Each black bar denotes an AcuA peptide identified by HDX-MS. **B**. Residue-specific HDX of AcuA, either without or in presence of 1 mM acetyl-CoA. **C**. The difference in residue-specific HDX of AcuA in presence of 1 mM acetyl-CoA and AcuA in isolation. **D**. HDX of selected representative AcuA peptides. Data represent the mean ± s.d. of n = 9 replicates (three protein preparations measured in technical triplicates) are displayed.

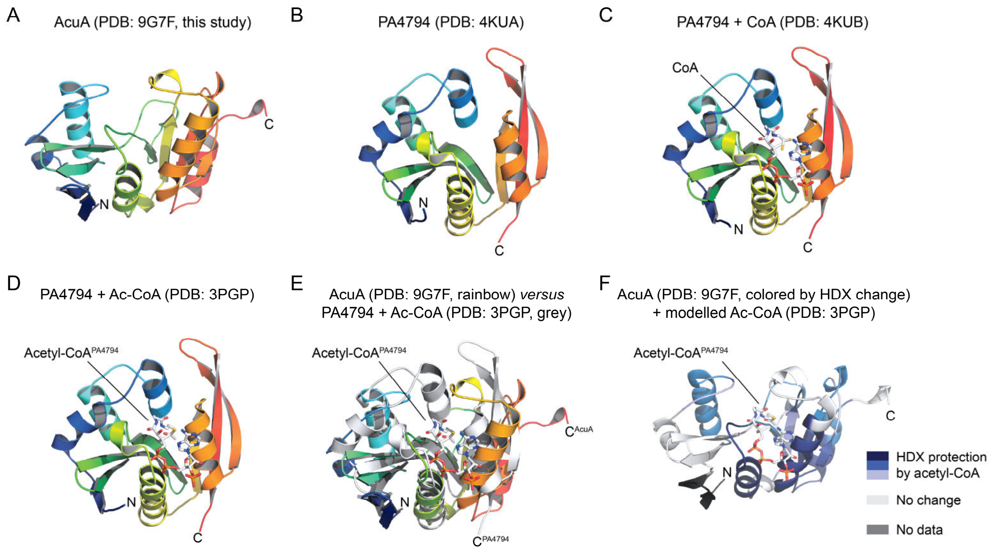

Supplementary Fig. 16. The acetyl-CoA binding pocket of AcuA compared to other GNAT proteins. A. Cryo-EM structure of AcuA derived from the AcuA/AcsA complex (PDB: 9G7F this study). **B-D.** Crystal structures of the GNAT superfamily acetyltransferase PA4794 [3], **B.** in absence of substrate (PDB: 4KUA), **C.** with CoA bound (PDB: 4KUB), and **D.** with acetyl-CoA bound (PDB: 3PGP). **E.** Superimposition of AcuA (rainbow from N to C-terminus) with PA4794 containing acetyl-CoA (grey). **F.** Changes in HDX of AcuA induced upon binding of acetyl-CoA. The highest difference at any HDX timepoint was projected onto the structure (compare to **Supplementary Fig. s10**). The position of the acetyl-CoA originates from the superimposition with acetyl-CoA-bound PA4794.

Supplementary Table 1. Statistics of data collection, structural refinement and model validation.

	AcsA-AcuA	AcsA dimer	AcsA-AcuA (CTD mask)	AcsA-AcuA (UA mask)
**Data collection**				
EM equipment	FEI Titan Krios			
Voltage (kV)	300			
Detector	K3			
Grid	UltrAuFoil 1.2/1.3			
Micrographs	6,152			
Particles for 3D classification	1,194,314			
Pixel size (Å)	0.708			
Defocus range (µm)	1.2-2.2			
Electron dose ($e^-/Å^2$)	50			
**Map refinement**				
Particles for refinement	124,994	169,773	27,521	49,819
Overall resolution of map (Å)	2.93	2.89	3.32	3.19
Map sharpening B-factor ($Å^2$)	-67	-93	-69	-61
**Model composition**				
Protein chains	3	2		
Protein residues	1198	898		
**Structural refinement**				
CC_mask	0.75	0.86		
CC_volume	0.73	0.84		
**Rms deviations**				
Bonds (Å)	0.004	0.003		
Angles (°)	0.740	0.586		
**Validation (protein)**				
Molprobity score	2.01	1.63		
Clashscore	18.49	9.04		
Good rotamers (%)	99.2	100%		
Ramachandran plot favored (%)	96.39	97.2		
Ramachandran plot allowed (%)	3.61	2.8		
Ramachandran plot outliers (%)	0.00	0.00		

Supplementary Table 2. Overview of data obtained by hydrogen/deuterium exchange mass spectrometry (HDX-MS).

	Experiment 1: AcuA/AcsA complex		Experiment 2: AcCoA binding to AcuA
Protein	AcuA	AcsA	AcuA
Conditions of HDX	25 °C in 20 mM HEPES-Na pH 7.5, 20 mM KCl, 20 mM $MgCl_2$, 200 mM NaCl; final D_2O = 90% (v/v)		
Time course of HDX	10/30/100/1,000/10,000 s		
Samples	1) AcuA (50 µM stock concentration/5 µM during HDX) 2) AcuA/AcsA complex (50 µM stock concentration/5 µM during HDX)	1) AcsA (50 µM stock concentration/5 µM during HDX) 2) AcuA/AcsA complex (50 µM stock concentration/5 µM during HDX)	1) AcuA (50 µM stock concentration/5 µM during HDX) 2) AcuA + AcCoA (50 µM stock concentration/5 µM during HDX + 10 mM stock concentration/1 mM during HDX)
Replicates	3 biological replicates (separate protein preparations) consisting of 3 technical replicates (separate HDX reactions) each		
Number of Peptides	85	202	117
Average peptide length (aa)	12.42	12.57	14.56
Sequence coverage (%)	90.9	96	87.2
Redundancy	5.31	4.55	8.92
Back-exchange	No correction for back-exchange based on a fully deuterated sample conducted		
Repeatability (average SD)	0.097 Da / 0.97%	0.093 Da / 0.88%	0.106 Da / 0.93%
Significance criterium applied	5% difference in relative HDX		

Supplementary Table 3. Plasmids used in this study.

Plasmids	Description
pPB167	pET-24d-Nhis-AcuA, a 633 bp fragment of *acuA* from gDNA of *Bacillus subtilis*, with BsaI inserted in pET-24d
pPB168	pET-24d-Chis-AcuA, a 633 bp fragment of *acuA* from gDNA of *Bacillus subtilis*, with BsaI inserted in pET-24d
pPB170	pET-24d-Chis-AcuB, a 645 bp fragment of *acuB* from gDNA of *Bacillus subtilis*, with BsaI inserted in pET-24d
pPB171	pET-24d-Nhis-AcuC, a 1164 bp fragment of *acuC* from gDNA of *Bacillus subtilis*, with BsaI inserted in pET-24d
pLZ005	pET-24d-Nhis-AcsA, a 1719 bp fragment of *acsA* from gDNA of *Bacillus subtilis*, with BsaI inserted in pET-24d
pLZ006	pET-24d-Chis-AcsA, a 1719 bp fragment of *acsA* from gDNA of *Bacillus subtilis*, with BsaI inserted in pET-24d
pLZ007	pGAT2-N-terminal GST-AcsA
pLZ008	pGAT2-N-terminal GST-AcuA
pLZ017	Mutation of K549A in pLZ005
pLZ202	pGAT2-N-terminal GST, deletion of 203 – 210 amino acids of AcuA in pLZ008
pLZ203	pGAT2-N-terminal GST, E85R of AcuA in pLZ008
pLZ204	pGAT2-N-terminal GST, E97R of AcuA in pLZ008
pLZ205	pGAT2-N-terminal GST, T134A of AcuA in pLZ008
pLZ206	pGAT2-N-terminal GST, Y136F of AcuA in pLZ008
pLZ207	pGAT2-N-terminal GST, R546E of AcsA in pLZ007
pLZ208	pGAT2-N-terminal GST, S547A of AcsA in pLZ007
pLZ258	pGAT2-N-terminal GST, E102Q of AcsA in pLZ008

Supplementary Table 4. **Primers used in this study.**

Primers	Sequence 5'-3'
pPB167-F	TTAAGGTCTCCCATGGGCCATCACCATCACCATCACGAACATCATAAAACATACCATTCAGC
pPB167-R	TTAAGGTCTCCTCGAGTTAATACATATAACGATGATAAAAACGGAGC
pPB168-F	TTAAGGTCTCCCATGGGCGAACATCATAAAACATACCATTC
pPB168-R	TTAAGGTCTCCTCGAGTTAGTGATGGTGATGGTGATGATACATATAACGATGATAAAAACGGAGC
pPB170-F	TTAAGGTCTCCCATGGGCATTGTTGAGCAAATCATGAAAAGAG
pPB170-R	TTAAGGTCTCCTCGAGTTAGTGATGGTGATGGTGATGTAGCAGATCCCTTTGCTCTG
pPB171-F	TTAAGGTCTCCCATGGGCCATCACCATCACCATCACAGAGACAGTGTATTTATCTATTCTCCATC
pPB171-R	TTAAGGTCTCCTCGAGTTACTTTGTTCTTTGCTGTTCAGAACG
pLZ005-F	TTAAGGTCTCCCATGGGCCATCACCATCACCATCACAACTTGAAAGCGTTACCAGC
pLZ005-R	TTAAGGTCTCCTCGAGTTAATCCTCCATTGTTGACAGATC
pLZ006-F	TTAAGGTCTCCCATGGGCAACTTGAAAGCGTTACCAGC
pLZ006-R	TTAAGGTCTCCTCGAGTTAGTGATGGTGATGGTGATGATCCTCCATTGTTGACAGATC
pLZ007-F	TTAAGGTCTCCCATGGGCAACTTGAAAGCGTTACCAGCAA
pLZ007-R	TTAAGGTCTCCTCGAGTTAATCCTCCATTGTTGACAGATC
pLZ008-F	TTAAGGTCTCCCATGGGCGAACATCATAAAACATACCATTCAGC
pLZ008-R	TTAAGGTCTCCTCGAGTTAATACATATAACGATGATAAAAACGGAGC
pLZ017-F	CCGAAAACCAGAAGCGGAGCAATCATGAGGCGCGTGCTG
pLZ017-R	CAGCACGCGCCTCATGATTGCTCCGCTTCTGGTTTTCGG
pLZ202-F	TTAAGGTCTCCCATGGGCGAACATCATAAAACATACCATTCAGC
pLZ202-R	TTAAGGTCTCCTCGAGTTAACGGAGCCTGTCAAATTGCTCA
pLZ203-F	ACCCGCTCCGGAGATGGTCTGAA
pLZ203-R	TTCAGACCATCTCCGGAGCGGGT
pLZ204-F	GAGGATTTGATTCGGCTCGGA
pLZ204-R	TCCGAGCCGAATCAAATCCTC
pLZ205-F	TGATGACGGCAGAATATTACTGG
pLZ205-R	CCAGTAATATTCTGCCGTCATCA
pLZ206-F	CAGAATTTTACTGGCATTGGGA
pLZ206-R	TCCCAATGCCAGTAAAATTCTG
pLZ207-F	CGAAAACCGAAAGCGGAAAGAT
pLZ207-R	ATCTTTCCGCTTTCGGTTTTCG
pLZ208-F	CGAAAACCAGAGCAGGAAAGATC
pLZ208-R	GATCTTTCCTGCTCTGGTTTTCG
pLZ258-F	CTCGGAGCCATCcAAGTAGCG
pLZ258-R	CGCTACTTgGATGGCTCCGAG

Supplementary references:

1 Gulick, A. M., Starai, V. J., Horswill, A. R., Homick, K. M. & Escalante-Semerena, J. C. The 1.75 A crystal structure of acetyl-CoA synthetase bound to adenosine-5'-propylphosphate and coenzyme A. *Biochemistry* **42**, 2866-2873, doi:10.1021/bi0271603 (2003).

2 Jogl, G. & Tong, L. Crystal Structure of Yeast Acetyl-Coenzyme A Synthetase in Complex with AMP. *Biochemistry* **43**, 1425-1431, doi:10.1021/bi035911a (2004).

3 Majorek, K. A., Kuhn, M. L., Chruszcz, M., Anderson, W. F. & Minor, W. Structural, functional, and inhibition studies of a Gcn5-related N-acetyltransferase (GNAT) superfamily protein PA4794: a new C-terminal lysine protein acetyltransferase from pseudomonas aeruginosa. *J Biol Chem* **288**, 30223-30235, doi:10.1074/jbc.M113.501353 (2013).
