# Supplementary material for: Regulation of acetyl-CoA biosynthesis via an intertwined acetyl-CoA synthetase/acetyltransferase complex
Source: Nat Commun. 2025 Mar 15;16:2557. doi: 10.1038/s41467-025-57842-2 (PMC11910552; doi:10.1038/s41467-025-57842-2)
Supplement: Supplementary file 2 — Reporting Summary [file 41467_2025_57842_MOESM2_ESM.pdf]

nature portfolio

Corresponding author(s): Zheng, Liujuan
Bange, Gert

Last updated by author(s): Feb 21, 2025

Reporting Summary

Nature Portfolio wishes to improve the reproducibility of the work that we publish. This form provides structure for consistency and transparency in reporting. For further information on Nature Portfolio policies, see our Editorial Policies and the Editorial Policy Checklist.

Statistics

For all statistical analyses, confirm that the following items are present in the figure legend, table legend, main text, or Methods section.

n/a	Confirmed	
☐	☒	The exact sample size (*n*) for each experimental group/condition, given as a discrete number and unit of measurement
☐	☒	A statement on whether measurements were taken from distinct samples or whether the same sample was measured repeatedly
☒	☐	The statistical test(s) used AND whether they are one- or two-sided<br>*Only common tests should be described solely by name; describe more complex techniques in the Methods section.*
☒	☐	A description of all covariates tested
☒	☐	A description of any assumptions or corrections, such as tests of normality and adjustment for multiple comparisons
☐	☒	A full description of the statistical parameters including central tendency (e.g. means) or other basic estimates (e.g. regression coefficient) AND variation (e.g. standard deviation) or associated estimates of uncertainty (e.g. confidence intervals)
☒	☐	For null hypothesis testing, the test statistic (e.g. *F*, *t*, *r*) with confidence intervals, effect sizes, degrees of freedom and *P* value noted<br>*Give P values as exact values whenever suitable.*
☒	☐	For Bayesian analysis, information on the choice of priors and Markov chain Monte Carlo settings
☒	☐	For hierarchical and complex designs, identification of the appropriate level for tests and full reporting of outcomes
☒	☐	Estimates of effect sizes (e.g. Cohen's *d*, Pearson's *r*), indicating how they were calculated

Our web collection on statistics for biologists contains articles on many of the points above.

Software and code

Policy information about availability of computer code

Data collection	Akta system: UNICORN 7 (Cytiva); cryo-EM: RELION-4.0.; HDX-MS: ProteinLynx Global SERVER (PLGS, Waters) and DynamX 3.0 softwares (Waters) .; Mass-Photometry: TwoMP mass photometer (Refeyn Ltd., UK), operated with the AcquireMP v2023 R1.1 software (Refeyn Ltd., UK).
Data analysis	Mass-Photometry: DiscoverMP v2023 R.1.2 software (Refeyn Ltd., UK).; Kinetics: GraphPad Prism (GraphPad Prism Corp., San Diego). Pymol Version 2.5.5 (Schroedinger); Coot Version 0.9.8.7 EL ; Phenix Version 1.17.1-3660 and 1.19; ChimeraX version 1.7.1; Microsoft Excel 2013 for Windows 10.;

For manuscripts utilizing custom algorithms or software that are central to the research but not yet described in published literature, software must be made available to editors and reviewers. We strongly encourage code deposition in a community repository (e.g. GitHub). See the Nature Portfolio guidelines for submitting code & software for further information.

Data

Policy information about availability of data

All manuscripts must include a data availability statement. This statement should provide the following information, where applicable:

- Accession codes, unique identifiers, or web links for publicly available datasets
- A description of any restrictions on data availability
- For clinical datasets or third party data, please ensure that the statement adheres to our policy

HDX-MS data have been deposited to the ProteomeXchange Consortium via the PRIDE37. Partner repository with the dataset identifier PXD058390 and are appended as Supplementary Table 2. The Cryo-EM data supporting the findings of this study have been deposited in the EMDB with deposition IDs 9G79 (https://www.rcsb.org/structure/unreleased/9G79) and 9G7F (https://www.rcsb.org/structure/unreleased/9G7F) . PDB-ID for other Acs including 1T5D (https://www.rcsb.org/structure/1T5D) and 1PG4 (https://www.rcsb.org/structure/1PG4) were downloading from PDB database.

Research involving human participants, their data, or biological material

Policy information about studies with human participants or human data. See also policy information about sex, gender (identity/presentation), and sexual orientation and race, ethnicity and racism.

Reporting on sex and gender	Not involved
Reporting on race, ethnicity, or other socially relevant groupings	Not involved
Population characteristics	Not involved
Recruitment	Not involved
Ethics oversight	Not involved

Note that full information on the approval of the study protocol must also be provided in the manuscript.

Field-specific reporting

Please select the one below that is the best fit for your research. If you are not sure, read the appropriate sections before making your selection.

☒ Life sciences ☐ Behavioural & social sciences ☐ Ecological, evolutionary & environmental sciences

For a reference copy of the document with all sections, see nature.com/documents/nr-reporting-summary-flat.pdf

Life sciences study design

All studies must disclose on these points even when the disclosure is negative.

Sample size	Experiments were performed with at least replicates.
Data exclusions	No data were excluded from consideration.
Replication	Figures 1c–e and 2h were conducted in replicates, with one representative result shown. Figure 1f was performed in triplicate, with one representative result displayed. Figure 3 and all panels in Figure 4 were conducted in biological triplicates.
Randomization	Randomization was not necessary for this study because the investigators were comparing well-characterized proteins under controlled conditions (e.g., addition of different ligands). No human or animal subjects were used in the study. Randomization is not typically employed in this field.
Blinding	Blinding was also not necessary, as the differences were strong and did not require subjective judgment or interpretation.

Reporting for specific materials, systems and methods

We require information from authors about some types of materials, experimental systems and methods used in many studies. Here, indicate whether each material, system or method listed is relevant to your study. If you are not sure if a list item applies to your research, read the appropriate section before selecting a response.

Materials & experimental systems

n/a	Involved in the study	
☐	☒	Antibodies
☒	☐	Eukaryotic cell lines
☒	☐	Palaeontology and archaeology
☒	☐	Animals and other organisms
☒	☐	Clinical data
☒	☐	Dual use research of concern
☒	☐	Plants

Methods

n/a	Involved in the study	
☒	☐	ChIP-seq
☒	☐	Flow cytometry
☒	☐	MRI-based neuroimaging

Antibodies

Antibodies used

Anti-Acetylated-Lysine antibody (Rabbit monoclonal, Merk: SAB5600275) at a concentration of 100 µg/mL and a dilution of 1:1500.
Anti-rabbit IgG, HRP-linked Antibody (Cell Signaling Technology, 7074S) at a concentration of 100 µg/mL and a dilution of 1:1500.

Validation

https://www.sigmaaldrich.com/DE/de/product/sigma/sab5600275, https://www.cellsignal.com/products/secondary-antibodies/anti-rabbit-igg-hrp-linked-antibody/7074?srsltid=AfmBOoqm7whiqecECmpFlt_j9QiMm12AdbUtwrckxdjuhxXUIiri_WE8

Plants

Seed stocks

Report on the source of all seed stocks or other plant material used. If applicable, state the seed stock centre and catalogue number. If plant specimens were collected from the field, describe the collection location, date and sampling procedures.

Novel plant genotypes

Describe the methods by which all novel plant genotypes were produced. This includes those generated by transgenic approaches, gene editing, chemical/radiation-based mutagenesis and hybridization. For transgenic lines, describe the transformation method, the number of independent lines analyzed and the generation upon which experiments were performed. For gene-edited lines, describe the editor used, the endogenous sequence targeted for editing, the targeting guide RNA sequence (if applicable) and how the editor was applied.

Authentication

Describe any authentication procedures for each seed stock used or novel genotype generated. Describe any experiments used to assess the effect of a mutation and, where applicable, how potential secondary effects (e.g. second site T-DNA insertions, mosiacism, off-target gene editing) were examined.
